# A Rare Case of Methamphetamine-Induced Severe Rhabdomyolysis and Compartment Syndrome

**DOI:** 10.7759/cureus.39804

**Published:** 2023-05-31

**Authors:** Nora Hajnoczky, Daniel George

**Affiliations:** 1 Internal Medicine, Einstein Healthcare Network, East Norriton, USA; 2 Nephrology, Einstein Healthcare Network, East Norriton, USA

**Keywords:** methamphetamine use, drug induced rhabdomyolysis, rhabdomyolysis with acute renal failure, acute kidney injury, compartment syndrome, methamphetamine-induced rhabdomyolysis

## Abstract

Mild cases of drug induced rhabdomyolysis are well documented, however severe cases require additional investigation. Here, we report a case of a 40-year-old female with no pertinent medical history who presented to the emergency department with bilateral leg weakness after recent polysubstance use. During the 26 days of hospitalization, the patient had three days of sustained creatine phosphokinase level of >42,000 U/L, oliguric acute renal failure treated with emergent dialysis, compartment syndrome requiring bilateral thigh and leg fasciotomies and required discharge to a long-term hemodialysis rehab center for ongoing management. The patient was diagnosed with a rare and life-threatening complication of methamphetamine (MA)-induced rhabdomyolysis. The relationship between MA-induced rhabdomyolysis and compartment syndrome is far from a novel concept. However, nearly all published cases demonstrate mild kidney injury and precipitating factors of agitated delirium and hyperpyrexia as the driving force for the compartment syndrome. In this report, we present a successfully treated, severe case of MA-induced kidney failure and rhabdomyolysis leading to compartment syndrome without clear indications of psychomotor agitation and hyperpyrexia. This report aims to highlight the importance of quick recognition of a rare methamphetamine side effect and the need for hasty response to limit complications and decrease hospital stay. Perhaps in the future, rhabdomyolysis etiology and severity may drive specific treatment plans.

## Introduction

Rhabdomyolysis and compartment syndrome have an intertwined relationship; the development of rhabdomyolysis and its treatment can cause compartment syndrome, and the expansion of compartment syndrome can exacerbate rhabdomyolysis. Rhabdomyolysis etiologies can be subdivided into traumatic and non-traumatic and further delineated by exertional or non-exertional forces [1]. Drug-induced rhabdomyolysis may cause direct muscle injury (i.e., drug myotoxicity) and indirect muscle injury through drug side effects (i.e., seizures or impaired circulation) [2,3]. A creatine phosphokinase (CPK) elevation of five times the upper limit of normal (>1000-1500 U/L) is a commonly accepted diagnostic indicator of rhabdomyolysis [4,5]. When CPK levels surpass 5000 U/L, muscle fiber cell death leads to increased circulating myoglobin and metabolites that can cause acute tubular obstruction, activation of reactive oxygen species and decreased vascular flow, resulting in acute kidney injury (AKI) [4-6]. At the forefront of treatment is fluid resuscitation; however, the severity, and rate of kidney failure progression may dictate the need for renal replacement therapy [6]. Thus, early recognition and close monitoring of electrolyte abnormalities and renal function are essential for survival. Additionally, after the diagnosis of rhabdomyolysis, the patient should be monitored for the development of compartment syndrome [6]. In the monitoring of compartment syndrome, the etiology of rhabdomyolysis and the patient’s clinical picture should be considered.

Here, we describe a case of a patient who presented with acute onset bilateral leg weakness; she was found to have rhabdomyolysis most likely secondary to methamphetamine (MA) use, which was complicated by renal failure, severe bilateral thigh and lower leg compartment syndrome, and an incidental pulmonary embolus.

## Case presentation

A 40-year-old female with a medical history of depression, anxiety, obesity, and polysubstance abuse was brought in to the emergency department (ED) by Emergency Medical Services (EMS) with altered mental status and acute bilateral lower extremity weakness. The patient stated that she had spent the prior night drinking alcohol and using marijuana. Prior to going to bed she had noticed bilateral leg weakness and was unable to walk up the stairs and get into bed. Per EMS, the patient was found at home, was hypotensive (80/30 mmHg) and received 500cc bolus of normal saline en route to the hospital, with resolution of blood pressure to 115/68 mmHg.

In the ED, vitals were significant for sinus tachycardia (100 beats/min), with temperature 35°C, physical examination positive for mild bilateral mottled feet, soft lower extremity compartments, no palpable dorsalis pedis or posterior tibial pulses bilaterally, faint right Doppler signal, and absent left Doppler signal, with a limited sensory and motor exam due to lack of patient cooperation. Pertinent laboratory results included potassium 6.1 mmol/L, creatinine 1.8 mg/dL, CPK 30,571 U/L, white blood cell count 25.8 K/uL, urine drug screen (UDS) positive for methamphetamine, fentanyl and benzodiazepines, and alcohol level within normal limits (Table 1). Bilateral lower extremity CT angiogram indicated diminutive caliber, but patent vasculature from iliac arteries to foot, and an incidental right lower lobe subsegmental pulmonary embolus was found. As the patient was rewarmed, pulses became palpable bilaterally. She was started on emergent hemodialysis due to worsening, sustained hyperkalemia >7 mmol/L, acute kidney injury likely from rhabdomyolysis, drug use and dye loading for CT imaging.

**Table 1:** Significant lab values before, during and after hospitalization K: potassium, Cr: creatinine, ALT: alanine transaminase, AST: aspartate transaminase, CKP: creatine phosphokinase, WBC: white blood cell

Lab	6 months earlier	Admission	Day 2	Day 3	Day 4	Day 5	Day of discharge	45 days post-discharge	Normal values
K (mmol/L)	3.8	6.1	5.3	5.4	5	4.5	5.1	2.8	3.5-5.2
Cr (mg/dL)	0.68	1.82	2.21	3.36	3.28	3.4	4.29	0.97	0.76-1.27
ALT (U/L)	31	151	1403	997	590	361	--	--	0-44
AST (U/L)	62	263	3644	1862	751	403	--	--	0-40
CPK (U/L)	--	30,571	>42,670	>42,670	>42,670	18,323	--	--	44-196
WBC (K/uL)	15.6	25.8	22.1	22.9	28.7	17.2	12.7	9.6	3.4-10.8

The following morning, the patient reported severe lower extremity pain. On examination, she had progressive edema, increased firmness in leg compartments, and tenderness to palpation, normal motor and sensory examination, +1 femoral pulses and dorsalis pedis Doppler pulse. Laboratory values were significant for creatinine 2.21 mg/dL, alanine transaminase 1403 U/L, aspartate transaminase 3644 U/L, CPK >42,670 U/L (maximum laboratory quantification) (Table 1). Bilateral venous ultrasound was negative for deep venous thrombi. The patient underwent bilateral three-compartment thigh fasciotomy and bilateral four-compartment lower leg fasciotomy.

She was treated with four consecutive days of hemodialysis for oliguria, and potassium and creatinine stabilization, followed by three maintenance sessions a week. For several days, the CPK remained elevated beyond 42,670 U/L (maximum laboratory quantification) until trending downward (Table 1). The patient was downgraded from the intensive care unit 10 days after admission, but had an extended stay in the hospital complicated by anemia secondary to fasciotomy site blood loss and required seven transfusions. Throughout the hospital stay, the patient’s blood pressure remained stable (95-120s/60s-80s mmHg), and did not require any vasopressors. Lactic acid levels did not exceed normal lab values (0.5-2.20 mmol/L), with the highest measurement being 1.52 mmol/L. Unfortunately, due to the severity of the compartment syndrome, only three of the 14 fasciotomy sites were closed during hospitalization. The patient was discharged to a long-term rehab with ongoing hemodialysis sessions.

The patient was last seen eight months following hospital discharge; her lab work had returned to normal, and she no longer required hemodialysis. All fasciotomy sites had healed well, including skin graft on the left lateral calf, and patient’s mobility was improving bilaterally.

## Discussion

Skeletal muscle breakdown and the ensuing calcium dysregulation, mitochondrial injury and reactive oxygen species release are the driving forces of muscle death. The most common clinical causes are traumatic crush injuries, ischemia, metabolic derangements, drug intoxication, hyperthermia, and endocrine abnormalities [1]. Drug-induced rhabdomyolysis often results from direct biochemical alterations and indirect psychoactive effects of drugs known as agitated delirium, including severe agitation, seizures, hyperthermia, and combativeness [1].

In a recent study, drug-induced rhabdomyolysis was attributed to cocaine in 22.9% of the cases, to amphetamine in 16.2%, and to cannabis in 15.8% cases [7]. Interestingly, the majority of the cases were of patients presenting with CPK <10,000 U/L (990/1013 patients), while the group labelled as severe, with CPK >10,000 U/L, consisted of only 23/1013 (2.3%) investigated cases [7]. Unfortunately, the severe group was not further delineated by drug type. From this study, we can appreciate that cocaine intoxication is a more frequent cause of drug-induced rhabdomyolysis than methamphetamine, and severe MA-induced rhabdomyolysis may be a rare finding. To our knowledge, little to no reports have examined the role of fentanyl alone in rhabdomyolysis. One case report documented the combined effects of cocaine and fentanyl inducing rhabdomyolysis causing bilateral brachial plexopathy, with CPK at 21,292 U/L, while another study examined a severe case of rhabdomyolysis in a patient with UDS positive for cannabinoid, fentanyl, methemphatemine and ethanol, with peak CPK at 1,000,000 U/L requiring ongoing regular hemodialysis [8,9]. There are a few reported rare cases of olanzapine and methoxphenidine causing rhabdomyolysis as well [10,11]. Apart from the above-mentioned publication, there are a limited number of studies on the patient population and presentation characteristics in severe MA-induced rhabdomyolysis.

MA is a neurotransmitter stimulant that releases and inhibits the re-uptake of serotonin, dopamine and noradrenaline resulting in increased energy and euphoria. The increased energy can indirectly lead to muscle damage through psychomotor agitation [2,3]. Direct skeletal muscle damage occurs with cellular stress via membrane damage, ATP depletion and mitochondrial dysfunction, leading to apoptosis and cell death of myofibers [3]. A recent 10-month study at the emergency department of the Princess Alexandra Hospital in Australia found that 22 of the 634 MA-intoxicated patients presented with AKI, elevated CPK and rhabdomyolysis (median CPK 2695 U/L). No patients required dialysis, and the median length of stay was 19 hours [12]. The researchers hypothesized that the majority of the kidney damage was likely secondary to volume depletion while intoxicated; however, they indicated that renal vasoconstriction, hyperthermia, pigment nephropathy and hemodynamic instability may have also played a role [12]. The short admission course is likely a good indicator of the limited impact the elevated CPK may have had on the kidneys.

A large-scale retrospective case study assessed MA- and non-MA-induced rhabdomyolysis (CPK >1000 U/L) cases that presented to the ED during 1992-1997 at a Level I trauma center. Of the 521 patients with rhabdomyolysis diagnosis on discharge, 387 were suspected to have rhabdomyolysis from a cause other than trauma, burns and infection [2]. Of the 387, 166 patients had a positive MA-positive UDS, 15 of 166 developed acute renal failure, five required temporary dialysis and one required permanent dialysis [2]. Similar proportions of kidney injury were observed in the non-MA-induced rhabdomyolysis group (primarily ethanol, cocaine, opiate users) [2]. However, there were statistical differences when it came to admission and length of hospital stay (MA, N=124; non-MA, N=188, p=0.001, MA 3.6 ± 2.6 days, non-MA 5.5 ± 3.8 days, p<0.010) [2]. Additionally, MA-induced rhabdomyolysis patients had a significantly higher initial CPK level than non-MA patients (12,439 ± 2404 U/L vs. 5,678 ± 566 U/L, p<0.02), while the peak CPK values were significantly greater in the non-MA group (16,827 ± 2,855 U/L vs. 19,426 ± 2,177 U/L, p<0.03) [2]. One of the previously mentioned studies by Waldman et al. demonstrated that the peak CPK value was found to positively correlate with a prolonged hospital stay [7]. These studies together may further reinforce that MA-induced rhabdomyolysis patients often have a more benign hospital course, and thus, kidney injuries and other complications requiring hospitalization are less severe than those induced by other drugs.

To our knowledge, there is only one other case report that presented with similar severe complications of MA intoxication rhabdomyolysis followed by acute renal failure and compartment syndrome. The patient was a 21-year-old male who had consumed MA from a water filtration pipe; he presented as febrile and hypotensive, with tachycardia, CPK levels in the 50,000s U/L, with elevated potassium and creatinine [13]. The patient’s CPK levels increased to 700,000s U/L during hospitalization, but he was successfully treated with continuous renal replacement for 20 days and fasciotomy for compartment syndrome [13]. This case may appear similar in severity caliber to our case; however, the other patient appeared to have more significant and substantial documented agitated delirium, hyperthermia, and potential seizures that may have been driving his CPK levels and thus causing oliguric kidney failure.

## Conclusions

MA is considered one of the common toxins that may cause rhabdomyolysis. However, compared to other rhabdomyolysis etiologies, MA appears to have a milder course, with shorter hospital stay, decreased duration of renal impairment and decreased need for hemodialysis. The MA-induced rhabdomyolysis case we present here is among the first reported with a high mortality risk, prolonged hospital course, severe AKI requiring more than a month of dialysis and bilateral lower extremity compartment syndrome. While the patient did have a one-time low blood pressure recorded per EMS, we do not believe it played a significant role in the patient's muscle damage as her blood pressure was normal on arrival to the hospital, her mean arterial pressure was greater than 65 mmHg during the majority of the hospitalization, she did not require vasopressor support and she had no rise in lactic acid levels during the hospitalization. At this point, it is unclear why our patient presented with a more severe clinical phenotype, and why she developed compartment syndrome without the usual causes (i.e., agitated delirium, hyperthermia, and seizures). However, we noted that the patient combined MA with other drugs (fentanyl, benzodiazepine) and perhaps the combination of these may have played a role. This may be an important subject for future studies.
